# Correction to “Enhancement of Blue Doping-Free and Hyperfluorescent Organic Light Emitting Diode Performance through Triplet–Triplet Annihilation in the Derivatives of Anthracene and Carbazole”

**DOI:** 10.1021/acsaelm.5c01365

**Published:** 2025-07-09

**Authors:** Oleksandr Bezvikonnyi, Audrius Bucinskas, Pavel Arsenyan, Alla Petrenko, Zheng-Yu Wei, Jiun-Haw Lee, Dmytro Volyniuk, Ehsan Ullah Rashid, Tien-Lung Chiu, Juozas Vidas Grazulevicius

In the original article, a grant number was mistakenly included in the Acknowledgments section. The correct Acknowledgments section is provided below. The corrections do not affect the investigation and the conclusions of the study.

**ACKNOWLEDGMENTS**: This work was supported by the Project of Scientific Cooperation Program between Latvia, Lithuania, and Taiwan (Grant LV-LT-TW/2023) and Research Council of Lithuania (LMTLT), Agreement No. S-LLT-22-4. The study has also received funding and support from the National Science and Technology Council (NSTC), Taiwan, under Grant NSTC 111-2923-E-155-002-MY3.

